# Our Popular Science Department

**Published:** 1881-05

**Authors:** 


Our Popular Science Department.—We take great pleasure in calling attention again to our new department, and feel fully assured it will receive the hearty endorsement of the hardly worked portion of the profession who have but little time at their command, and cannot peruse a moiety of the journals now published on medicine and the collateral sciences, and yet who like to know as much as their limited time will permit of what is passing in the scientific world. The articles will be as concise as they can be made, to retain their interest and usefulness. An original article was promised for this department, but it has not yet come to hand. Brief original papers will, however, be introduced from time to time, as they may be thought necessary, and we beg leave to again solicit such from friends who may be able to obtain sufficient leisure to prepare them for our pages.

